# Mutation analysis of CBP and PCAF reveals rare inactivating mutations in cancer cell lines but not in primary tumours

**DOI:** 10.1038/sj.bjc.6600554

**Published:** 2002-11-04

**Authors:** H Özdağ, S J Batley, A Försti, N G Iyer, Y Daigo, J Boutell, M J Arends, B A J Ponder, T Kouzarides, C Caldas

**Affiliations:** Cancer Genomics Program, Department of Oncology, University of Cambridge, Hutchison/MRC Research Centre, Cambridge CB2 2XZ, UK; Wellcome/CRC Institute and Department of Pathology, University of Cambridge, Tennis Court Road, Cambridge CB2 1QR, UK; Molecular Histopathology, University of Cambridge Pathology Department, Addenbrooke's Hospital, Box 235, Level 3, Hills Road, Cambridge CB2 2QQ, UK

**Keywords:** P300, CBP, PCAF, mutations, epithelial cancers

## Abstract

In this study we screened the histone acetyltransferases CBP and PCAF for mutations in human epithelial cancer cell lines and primary tumours. We identified two CBP truncations (both in cell lines), seven PCAF missense variants and four CBP intronic microdeletions. These data suggest that neither gene is commonly inactivated in human epithelial cancers.

*British Journal of Cancer* (2002) **87**, 1162–1165. doi:10.1038/sj.bjc.6600554 www.bjcancer.com

© 2002 Cancer Research UK

## 

The addition of an acetyl group to specific lysine residues within the N-terminal region of the four core histone proteins by acetyltransferases, causes the destabilisation of the chromatin structure and enhances access of transcription factors and other DNA-binding components to DNA (Grunstein, 1997). The histone acetyltransferases CBP, P300 and PCAF also acetylate sequence-specific transcription factors such as P53. CBP was originally isolated on the basis of its interaction with CREB in response to cAMP signalling (Chrivia *et al*, 1993). P300 was purified as a cellular protein, which binds the adenoviral protein E1A (Eckner *et al*, 1994). PCAF, P300/CBP-associated factor, was the first mammalian histone acetyltransferase discovered on the basis of homology to yeast Gcn5p (Yang *et al*, 1996). The fact that histone acetyltransferases are involved in cell proliferation and differentiation suggests that they may be involved in cancer. Indeed *P300* (also known as *EP300*) and *CBP* are fused to *MLL* in acute myeloid leukaemia. It is also known that P300, CBP and PCAF are targeted by viral oncoprotein E1A (Eckner *et al*, 1994; Yang *et al*, 1996; Chakravarti *et al*, 1999). In colorectal and gastric carcinomas two somatic *P300* missense mutations coupled to deletion of the second allele of the gene were identified (Muruoka *et al*, 1996). The role of *P300* as a tumour suppressor gene was later confirmed with the identification of truncating, insertion and missense mutations in primary tumours and cancer cell lines, associated with inactivation of the second allele (Gayther *et al*, 2000). In this study we screened the whole coding sequence and intron–exon boundaries of both *CBP* and *PCAF* for somatic mutations in a series of human primary tumours and cancer cell lines. We also screened a panel of cell lines for truncating *P300* mutations using Western blotting.

## MATERIALS AND METHODS

### Samples

The *CBP* gene was screened in 179 DNA samples isolated from 59 primary breast tumours, 37 primary ovarian tumours, 20 colorectal tumours, and 63 cancer cell lines. The *PCAF* gene was screened in 80 cancer cell lines (31 breast, 25 ovarian, 10 pancreatic, 6 SCLC, 5 colorectal, 1 NSCLC, 1 MISC, 1 BCLL) and 20 primary colorectal tumours. In all cases the collection of tumour material was done with Local Research Ethics Committee approval. All tumours were ‘flash’ frozen immediately following surgery. Cell lines were obtained from ATCC and ECACC cell repository or as a gift from collaborating laboratories.

### Preparation of DNA and RNA

Frozen primary tumours were serially sectioned onto slides. Tumour tissue was microdissected and DNA extracted by SDS-proteinase K digestion followed by phenol-chloroform extraction. Germ-line DNA was prepared from either a matching blood sample or from normal tissue. Cell line DNA was extracted by either proteinase K or DNAzol™ (Gibco BRL). RNA was extracted with TriZol™ (Gibco BRL). cDNA was synthesized by reverse transcription of RNA using random hexamers and Superscript II (Gibco BRL).

### Determination of the exon–intron structure of *CBP* and *PCAF*

The exon-intron structure of *CBP* and *PCAF* were determined from the available cDNA and genomic DNA sequences in Genbank (NCBI). *CBP* is a 8694 bp cDNA consisting of 32 exons distributed over 154 Kb of genomic sequence at chromosome band 16p13.3. PCAF is a 2957 bp cDNA consisting of 20 exons spread over 114 Kb of genomic sequence at chromosome band 3p24.

### Polymerase chain reaction

*CBP* was amplified from gDNA in 43 fragments and *PCAF* was amplified from cDNA in 13 fragments of approximately 200–400 bp (oligonucleotide primer sequences are available on request, ho212@cam.ac.uk). *PCAF* sequence alterations were confirmed subsequently in genomic DNA. Amplification reactions (30 μl) contained 20 mM (NH_4_)_2_SO_4_, 75 mM TrisHCl, pH 9.0 at 25°C, 0.1% (w v^−1^) Tween, 2.5–3 mM MgCl_2_, 200 μM dNTP, 10 pmoles of each primer and 2.5 U of Red Hot DNA polymerase (Advanced Biotechnologies). The amplifications were done using a DNA Engine Tetrad, MJ Research PTC-225 Peltier Thermal Cycler.

### Protein truncation test

*PCAF* coding sequence was analysed initially by PTT. Cell lines HCT15 and OVCAR8, which showed an altered sized P300 protein on Western blot were also analysed by PTT. RT–PCR amplification was done in overlapping fragments of approximately 1000–1200 bp in length each, using a 5′ oligo containing the appropriate sequences (oligonucleotide sequences are available on request). PTT reactions were performed following the manufacturer's protocol (Promega). Alterations found in PTT were confirmed by sequencing.

### SSCP/HA (Single Strand Conformation Polymorphism/Heteroduplex Analysis)

Formamide loading buffer was added to PCR products. The mix was denatured at 95°C for 10 min and kept on ice until loading onto 0.8×MDE (Mutation Detection Enhancement) gel (Flowgen), both with and/or without 10% Glycerol. Gels were run overnight at 120 V and 4°C.

### Western blot analysis

Western blot analysis was used to screen for *P300* truncating mutations in a panel of 24 cell lines. We also performed Western blot in cell lines identified to have truncating *CBP* mutations. Cell extracts were prepared by direct lysis on cell culture plates (TBS, 0.5% NP-40, 5 mM EDTA, Complete Protease Inhibitor Coctail, Boehringer), then electrophoresed in pre-cast polyacrylamide Tris-Glycine gels (Novex). The separated proteins were transferred to nitrocellulose membrane (Millipore) and hybridised with the respective primary (CBP A-22 Santa Cruz, P300 N-15 Santa Cruz) and secondary antibodies (Dako). Detection employed the ECL kit (Amersham).

### DNA Sequencing

Purified PCR products were sequenced using ABI Prism^R^ BigDye terminators and an ABI377 sequencer or ABI3100 genetic analyzer (Applied Biosystems, Foster, CA, USA). All samples with a mutation were re-amplified and re-sequenced.

## RESULTS AND DISCUSSION

### *CBP* mutations

Two different *CBP* truncating mutations were identified in the 63 cell lines analysed (Table 1

**Table 1:**
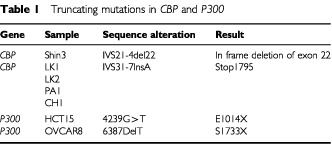
Truncating mutations in *CBP* and *P300*

). Shin3, an ovarian cancer cell line, was found to have a heterozygous 22 bp deletion in intron 21 at position −4 (Figure 1A

**Figure 1:**
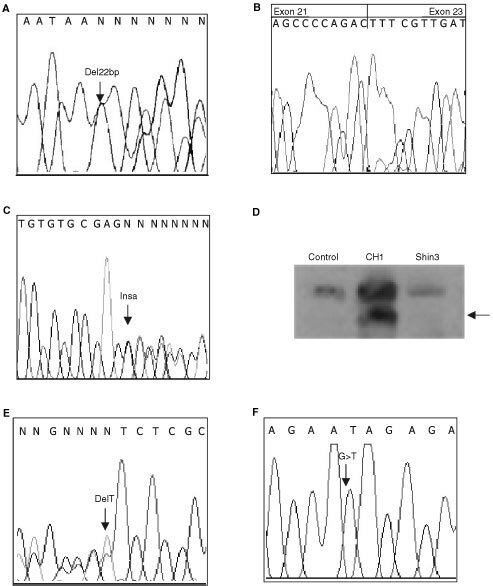
Truncating mutations in CBP and P300. (**A**) Sequencing chromatogram of Shin3 gDNA showing *CBP* IVS21-4del22 causing in frame deletion of exon 22. (**B**) Sequencing chromatogram of Shin3 cDNA showing in frame deletion of *CBP* exon 22. (**C**) Sequencing chromatogram of CH1 showing *CBP* IVS31-7InsA (Stop 1795). (**D**) Western blot analysis of CBP in CH1 showing truncated protein (Arrow). (**E**) Sequencing chromatogram of Ovcar8 (reverse strand) showing *P300* 6387delT (Stop1733). (**F**) Sequencing chromatogram of HCT15 showing *P300* 4239 G>T (Stop1014).

). This intronic deletion was shown to cause an in-frame deletion of the whole exon 22 at the cDNA level (Figure 1B). In four cancer cell lines (LK1, LK2, PA1, CH1) an identical heterozygous insertion of an A was found in intron 31 at position −7 (Figure 1C). This insertion was shown to create an alternative splice donor site. This in turn caused a frame-shift and a premature stop codon at nucleotide 5457 (codon 1795). This heterozygous mutation was confirmed using Western blotting (Figure 1D). The finding of the identical mutation raised the suspicion of cross contamination between cell lines. HLA typing was performed and the results showed that these cell lines were indeed the same, despite originating from two different labs (data not shown). We considered these as a single cell line for purposes of mutation frequency analysis and therefore the truncating mutations were identified in two distinct cell lines out of 60 analysed (3%). No truncating mutations were identified in 116 primary tumours. Small intronic microdeletions in *CBP* were identified in four samples (Table 2

**Table 2:**
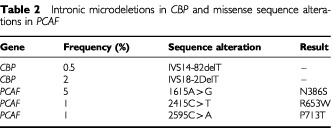
Intronic microdeletions in *CBP* and missense sequence alterations in *PCAF*

). A primary colorectal cancer had a heterozygous deletion of T at position −2 in intron 18. This tumour had no molecular phenotype suggestive of microsatellite instability (MSI). Two cancer cell lines with MSI, OVIP and HCT15, had an identical microdeletion. These intronic microdeletions, which were very close to the splice donor site, had no apparent effect on mRNA splicing as tested by amplification of cDNA with primers flanking exon19. A breast cancer cell line, MT3, was found to have a deletion of T in intron 14 at position −82, with no apparent effect on splicing as tested by RT–PCR. This cell line is also MSI+. Uncommon *CBP* single nucleotide polymorphisms were also detected (Table 3

**Table 3:**
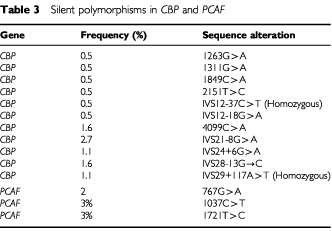
Silent polymorphisms in *CBP* and *PCAF*

).

Although we have characterised two truncating *CBP* mutations in cancer cell lines, the absence of inactivating mutations in the primary tumours analysed prevents us from unequivocally establishing the role of *CBP* in human primary cancers. Nevertheless the uncommon mutations identified, together with the increased tumour incidence in Rubinstein-Taybi syndrome (Petrij *et al*, 1995) and the tumorigenic phenotype in CBP mice (Kung *et al*, 2000) provide circumstantial evidence for a possible role of *CBP* as a tumour suppressor gene. We can only speculate on the significance of the intronic microdeletions seen, but we note that we have previously identified similar intronic microdeletions in other genes in MSI cell lines and primary tumours (data not shown).

### Missense PCAF sequence alterations

No truncating mutations were identified in the *PCAF* gene. We used cDNA to screen for truncating mutations and therefore nonsense mediated RNA decay (Maquat, 1995) could have contributed to a lower sensitivity of the mutation screen. Missense sequence alterations in PCAF were identified in 1 out of 20 primary tumours (5%) and 5 out of 80 cell lines (6%). In a colorectal cancer case the missense variant was a C to A transversion at nucleotide 2595 in exon 17, resulting in a proline to threonine substitution at codon 713. The same sequence alteration was also found in the germ-line DNA of the same individual (Table 2). The functional significance of this single nucleotide polymorphism was not tested but this residue is conserved in mouse and human GCN5, suggesting it might be important for protein function. Sequence analysis in DNA extracted from laser capture microdissected normal and tumour tissue samples confirmed the heterozygous mutation in both tumour and germ-line DNA, and therefore the mutation is not associated with somatic allelic deletion. A missense alteration at nucleotide 1615 (N386S) was found in four cell lines (Ovmana, Hela, L23, MC4000/ Matu). The mouse homologue of this residue is serine, implying that this alteration is a polymorphism. An ovarian cancer cell line, OVI-P, had a C to T transition at nucleotide 2415 resulting in Arg653Trp substitution. This arginine residue is conserved in human GCN5, and therefore this substitution could impair protein function. In addition to these missense sequence alterations a single nucleotide deletion in the 3′ untranslated region of *PCAF* was found in a colon cancer cell line, SW48. Three silent *PCAF* polymorphisms were also identified (Table 3).

The missense sequence alteration at nucleotide 1615 has been previously reported and considered a polymorphism (Nishimori *et al*, 2000).

### Truncating *P300* mutations

We have previously shown that truncating mutations resulted in the production of stable protein, detectable by Western blot. Using this approach we studied a panel of 24 cell lines and in two (8.3%) truncated protein was detected (Table 1). OVCAR8 had two bands on the Western blot, one of normal size and one from a truncated protein, suggesting a heterozygous mutation. Sequencing confirmed a heterozygous frameshift deletion (6387delT) resulting in truncation of the protein at codon 1733 (Figure 1E). HCT15 expressed only a truncated protein and sequencing showed a homozygous transversion, 4239 G→T, generating a stop codon (Figure 1F). This same mutation was previously found in DLD1 as a heterozygous alteration. Since it is known that DLD1 and HCT15 were derived from the same patient (Chen *et al*, 1995), one can interpret this result as indicating that the two cell lines were derived from different areas of the cancer or that the genetic progression represents an event occuring during *in vitro* culture.

The finding of truncating P300 mutations was not a surprise, since we have previously shown that P300 mutations occur in a small percentage of human epithelial malignancies and others have also identified truncating mutations (Oshima *et al*, 2001). We have now analysed a total of 222 cancer samples and truncating mutations have been detected in 6 out of 107 (5.6%) cell lines and 2 out of 115 (1.7%) primary tumours.

In conclusion, this report revealed inactivating CBP mutations in cancer cell lines but not in primary tumours. It also identified a few missense alterations in PCAF and intronic sequence variants in CBP. These data are insufficient to establish a role for either gene in epithelial cancers.
